# Multiple Giant Coronary Artery Aneurysms With a Coronary Pulmonary Artery Fistula: A Case Report

**DOI:** 10.7759/cureus.85328

**Published:** 2025-06-04

**Authors:** Yosuke Motoharu, Satoru Arai, Ryuji Hojo, Eiichi Teshima, Ryuji Tominaga

**Affiliations:** 1 Cardiothoracic Surgery, Fukuoka Wajiro Hospital, Fukuoka, JPN; 2 Cardiovascular Center, Kawakita General Hospital, Tokyo, JPN; 3 Cardiovascular Surgery, Fukuoka Wajiro Hospital, Fukuoka, JPN

**Keywords:** cardiopulmonary bypass, coronary artery fistula, giant coronary artery aneurysm, median sternotomy, on pump beating

## Abstract

Coronary artery aneurysms (CAAs) are rare. Giant CAAs are defined as those exceeding 2 cm in diameter. An 82-year-old woman was referred to our institution for surgery after coronary 3D computed tomography (CT) and transthoracic echocardiography (TTE) revealed CAAs and a coronary-to-pulmonary artery fistula. Coronary angiography identified four aneurysms, ranging from 10 to 45 mm in diameter, in the right coronary artery (RCA), left anterior descending (LAD) artery, and left circumflex (LCx) artery. The Qp/Qs ratio was 1.34. The surgical approach involved median sternotomy and was performed under cardiopulmonary bypass (CBP) on a beating heart. The aneurysms originating from the RCA and LAD artery measured 30 and 45 mm, respectively, and were easily visible. After isolating the feeding vessels, the aneurysms were opened, and their inflow and outflow vessels were sutured closed. Additionally, the feeding vessels were ligated at their origins, and the aneurysm walls were sutured. The locations of the small aneurysms were confirmed using direct echocardiography and treated similarly. There were no signs of damage to the normal coronary arteries, and revascularization was not required. The fistula was closed after opening the pulmonary artery. The patient was weaned off CBP without difficulty, and her postoperative course was uneventful. Postoperative 3D CT confirmed the disappearance of blood flow in the aneurysms and abnormal vessels. We report a case of multiple CAAs with complex feeding vessels that were successfully treated on a beating heart without revascularization.

## Introduction

Coronary artery aneurysm (CAA) is defined as a localized dilation exceeding 1.5 times the diameter of a normal coronary artery, with an incidence rate of 0.3%-4.9% among patients undergoing coronary angiography [1]. Giant CAAs, defined as those exceeding 2 cm in diameter, have an extremely low prevalence of 0.02%. CAAs most commonly occur in the right coronary artery (RCA), followed by the left anterior descending (LAD) artery, left circumflex (LCx) artery, and left main coronary artery (LMCA) [2,3]. The primary cause of CAA is atherosclerosis; however, other causes include trauma, Kawasaki disease, vasculitis, connective tissue disorders, congenital anomalies, infections, and iatrogenic factors [4]. Most CAAs are asymptomatic, and their natural history and prognosis remain unclear. Nevertheless, they pose clinical risks, such as thromboembolic complications from intraluminal thrombosis, potentially leading to myocardial infarction and aneurysm rupture - a serious concern [5].

## Case presentation

**Figure 1:**
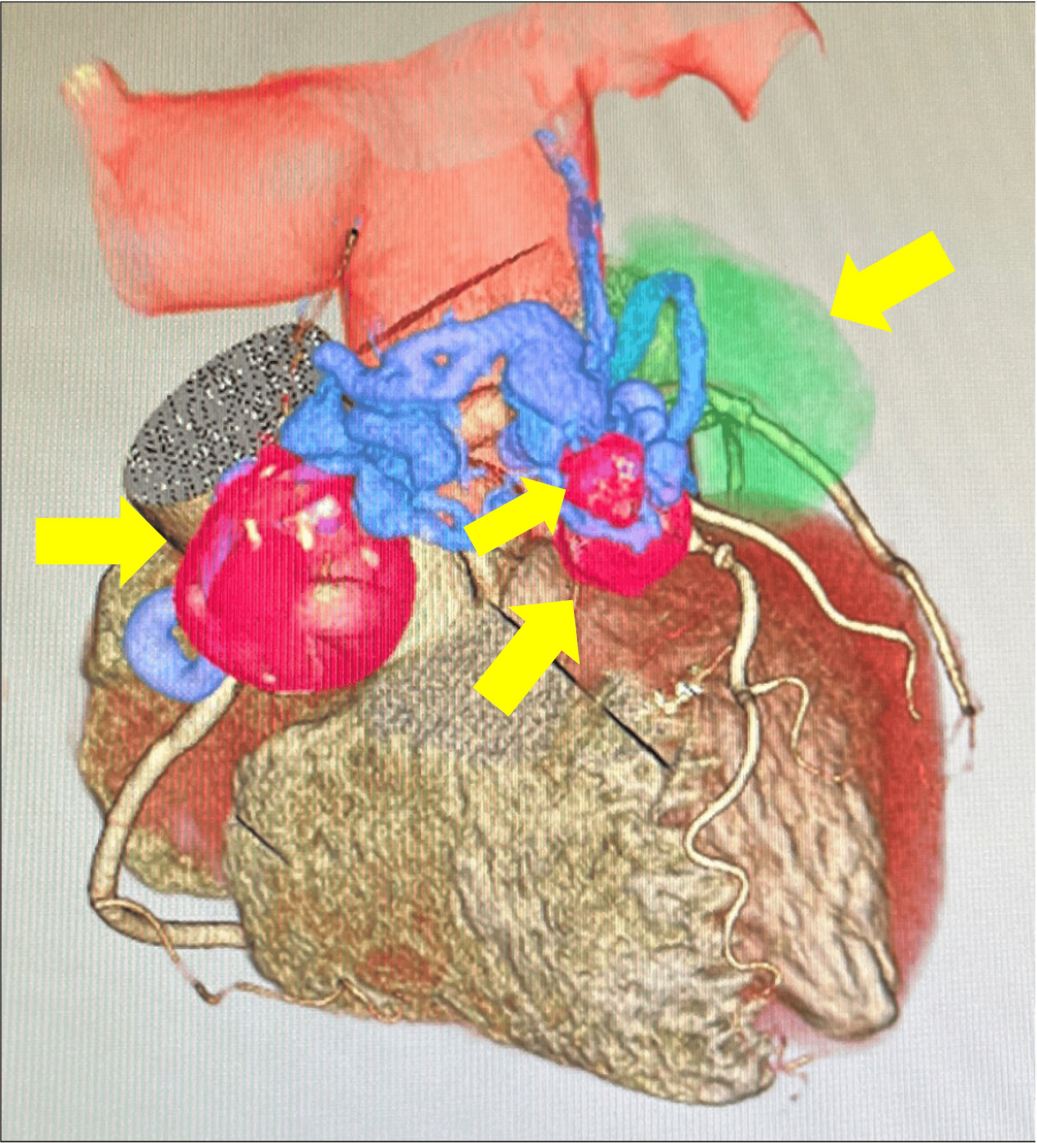
Preoperative coronary 3D CT image Yellow arrows indicate aneurysms. The largest aneurysm, colored green, has an aneurysm diameter of 45 mm. CT, computed tomography

An 82-year-old woman had been receiving oral treatment at a local hospital for non-tuberculous mycobacterial disease. She presented with cervical swelling and was diagnosed with lymphoma by the Hematology Department, prompting a full-body evaluation with computed tomography (CT) and echocardiography. Coronary 3D CT revealed four CAAs, ranging from 10 to 45 mm in diameter. One aneurysm, originating from the RCA, was supplied by an abnormal vessel arising from Segment 2, forming a 30 mm aneurysm on the anterior surface of the right ventricle. This vessel meandered over the anterior surface of the pulmonary artery, creating a fistula just distal to the pulmonary valve (Figure 1). Another aneurysm, measuring 18 mm, originated from an abnormal vessel at LAD Segment 6, coursing alongside the pulmonary artery, merging with the abnormal vessel from the RCA, and draining into the pulmonary artery. The largest aneurysm, measuring 45 × 37 mm, arose from a branch of the LCx Segment 11, and contained numerous thrombi. Additionally, a smaller, 18 mm aneurysm was detected downstream of the thrombus-filled aneurysm.

**Figure 2:**
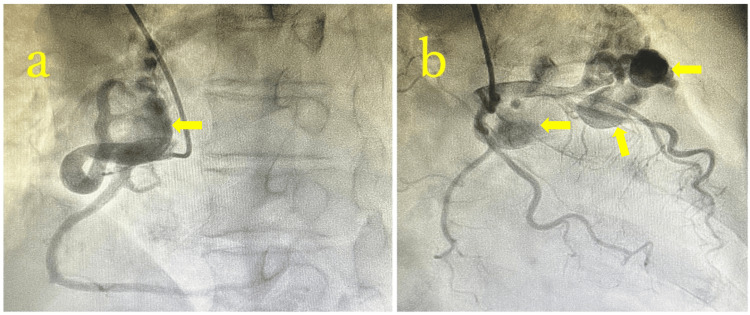
Preoperative coronary angiography Preoperative coronary angiography reveals an aneurysm arising from Segment 2 of the right coronary artery and a pulmonary artery fistula (arrow) (a). Aneurysms originating from abnormal vessels arising from the LAD and LCx were observed (arrows) (b). LAD, left anterior descending artery; LCx, left circumflex artery

Preoperative transthoracic echocardiography (TTE) revealed a left ventricular diastolic/systolic diameter (Dd/Ds) of 48/32 mm and a left ventricular ejection fraction (LVEF) of 62%, with no regional wall motion abnormalities or significant valvular disease. Coronary angiography demonstrated that all aneurysms were supplied by abnormal vessels, with no significant stenosis or obstruction in the normal coronary arteries, which were well visualized at their distal ends (Figure 2). Swan-Ganz catheterization revealed a Qp/Qs ratio of 1.34.

Operative technique

**Figure 3:**
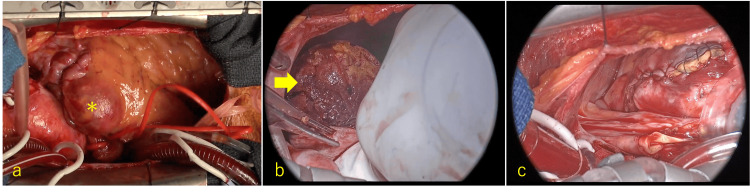
Intraoperative findings Intraoperative findings revealed an abnormal vessel originating from the taped Segment 2 and a giant CAA (asterisk) (a). The largest aneurysm was filled with thrombus (arrow), which was removed en bloc (b). The inflow and outflow vessels were sutured and ligated from the inside, and the aneurysmal wall was sutured (c). CAA, coronary artery aneurysm

The surgical technique is demonstrated in Video 1. The surgical approach involved median sternotomy. To manage both the inflow and outflow vessels of the aneurysms, the operation was conducted on a beating heart using cardiopulmonary bypass (CPB) via cannulation of the ascending aorta and bicaval cannulation. A left ventricular vent was inserted through the right superior pulmonary vein. Aneurysms from the RCA and LAD, measuring 30 mm and 45 mm, respectively, were easily visible (Figure 3a). The abnormal vessel from the RCA was ligated. After confirming backflow from both the inflow and outflow vessels, they were closed with 5-0 polypropylene sutures. The inflow vessel was also ligated at its origin, and the aneurysm wall was sutured closed. Identifying inflow vessels for the other aneurysms was challenging, with a high risk of damaging normal coronary arteries during dissection. Therefore, the aneurysm walls were opened, and inflow and outflow vessels were sutured from the inside. The meandering abnormal vessels surrounding the aneurysms were sutured extensively. The largest aneurysm, measuring 45 mm and filled with thrombus, was treated with en bloc removal, followed by suturing of the inflow and outflow vessels using 5-0 pledgeted polypropylene (Figure 3b). The aneurysm wall was closed and sutured (Figure 3c). An additional 10 mm aneurysm, receiving blood flow from both the LAD and LCx, was identified using direct echocardiography and treated similarly. The coronary artery-to-pulmonary artery fistula was addressed by making a transverse incision in the abnormal vessel along the base of the pulmonary artery. A 5 mm fistula was identified and closed from the inside using 5-0 polypropylene. Direct echocardiography confirmed the absence of blood flow to the aneurysm and fistula.

**Video 1:** Aneurysmectomy for multiple giant coronary artery aneurysms with on-pump, beating-heart technique By incising the aneurysms before ligating the inflow vessels, both the inflow and outflow vessels can be easily controlled from within the aneurysmal lumen, thereby minimizing unnecessary dissection.

**Figure 4:**
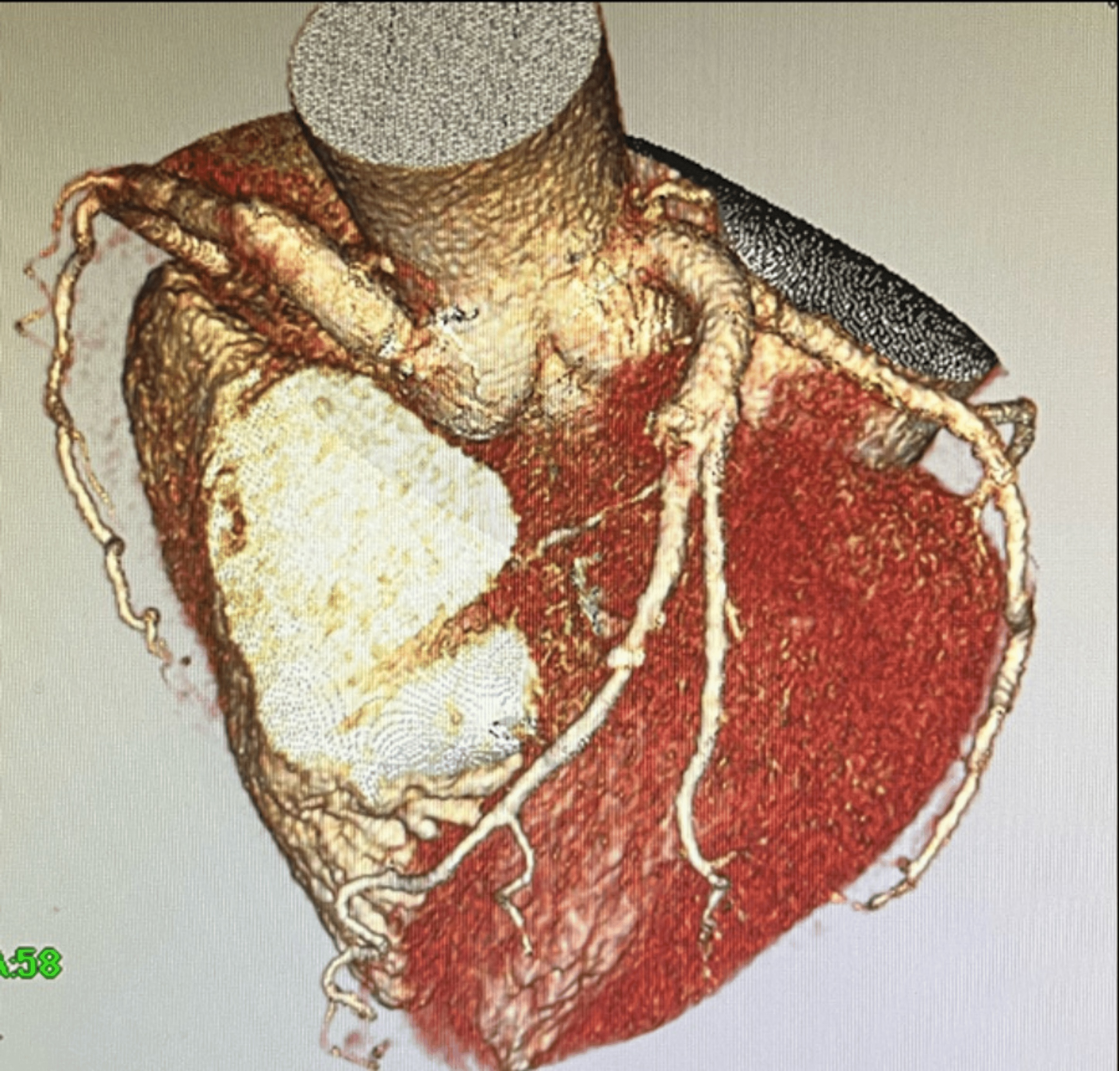
Postoperative coronary 3D CT image Postoperative coronary CT revealed the disappearance of the coronary artery aneurysms, abnormal vessels, and the coronary artery-to-pulmonary artery fistula. CT, computed tomography

No significant changes in ST segments were observed on the intraoperative 12-lead electrocardiogram (ECG), and no wall motion abnormalities were noted on transesophageal echocardiography, making coronary artery bypass grafting unnecessary. The total operative time was 223 minutes, with CPB lasting 159 minutes. Postoperative coronary CT confirmed the disappearance of the aneurysms, abnormal vessels, and fistulas, with no stenosis or occlusion in the normal coronary arteries (Figure 4). The patient was discharged on postoperative day 12 without any complications.

## Discussion

Underlying conditions associated with CAA include genetic predisposition in idiopathic cases, overexpression of specific enzymes in atherosclerosis, and autoimmune and inflammatory processes seen in Kawasaki disease, Takayasu’s arteritis, and Marfan syndrome. Other contributing factors include dynamic changes in wall stress due to cocaine abuse, iatrogenic damage, and infections [6]. Reports of coronary artery fistulas (CAFs) complicated by CAAs, as observed in this case, are relatively uncommon. CAF is a congenital abnormality where part of a coronary artery opens abnormally into the pulmonary artery or a cardiac chamber, affecting 0.2%-0.4% of patients with congenital heart disease [1]. Many CAAs are incidentally detected through CT, echocardiography, or coronary angiography, posing risks such as heart failure from left-to-right shunting or ischemia due to the “coronary steal” phenomenon. The mechanisms for CAA development associated with CAF likely involve the inherent fragility of the abnormal vessels, along with factors such as tortuosity, stenosis, turbulence, inflammation, and atherosclerosis.

In this case, despite multiple large CAAs arising from all three major coronary arteries, the normal coronary arteries supplying the myocardium showed no stenotic or dilative lesions due to atherosclerosis. This finding suggests that the abnormal vessels forming the fistula are more fragile and prone to dilation than normal coronary arteries. The most significant limitation of this report is that all of the incised CAFs and CAAs were closed by suture, leaving no specimens available for pathological evaluation.

The criteria for surgical intervention for CAA remain unclear. Baman et al. reported an association between CAA and increased five-year mortality (adjusted hazard ratio: 1.56; 95% confidence interval: 1.01 to 2.41) [7]. Konno and Endo proposed the following indications for surgery in cases of CAF [8]: (1) shunt ratio exceeding 30%, (2) evidence of ischemia or cardiac overload on electrocardiography, (3) progressive pulmonary hypertension or congestive heart failure, (4) a saccular aneurysm at risk of rupture, (5) a history of bacterial endocarditis, and (6) social impact due to heart murmurs. Sekine et al. suggested surgical intervention for: (1) symptomatic cases, (2) shunt ratio (Qp/Qs) ≥1.5 or ≥30%, and (3) aneurysm diameter exceeding 30 mm, with symptoms being a primary criterion [9]. However, reports exist of ruptured aneurysms measuring <30 mm, indicating that surgical decisions should consider not only aneurysm size but also morphology, the presence of ischemia or heart failure, and any history of infection.

In this case, the decision for surgery was based on the presence of a coronary fistula with a shunt ratio of 30%, giant CAA, and multiple saccular aneurysms. Although percutaneous coronary intervention (PCI) with coils or covered stents has shown favorable outcomes, particularly in cases of acute coronary syndrome, this case involved lesions extending across multiple coronary branches, making surgical intervention the preferred approach [6]. Furthermore, in cases where the coronary lesions are not associated with inflammatory conditions such as Kawasaki disease, repeated interventions may be required following PCI, and current American Heart Association guidelines do not recommend PCI [10]. 

Performing surgery for CAAs under CPB with the heart beating is not necessarily a remarkable technique. In surgical treatment for CAAs, the most important factors are: ensuring that no residual blood flow remains within the aneurysm, preventing the thrombus inside the aneurysm from embolizing to the distal coronary artery, and avoiding damage to the normal coronary artery. In cases of multiple CAAs associated with CAF and complex abnormal vessels, such as in this case, identifying and ligating all inflow and outflow vessels can be challenging. Therefore, opening the aneurysm without dissecting the surrounding tissue or applying vessel taping, and suturing the inflow and outflow vessels from within, is a more feasible approach. Performing the procedure on a beating heart reduces the risk of thrombus embolization into distal vessels by maintaining blood flow. When performing surgery under CPB, there is no concern about bleeding or hemodynamics, and rapid intervention can be made in response to any flow disturbances in the normal coronary artery during surgery, as detected by intraoperative electrocardiography or transesophageal echocardiography.

## Conclusions

We successfully treated a case of multiple giant CAAs associated with a CAF through aneurysm excision and fistula closure, without revascularization, using CPB on a beating heart. Although this technique is one of the standard approaches, it is considered the most useful due to its ability to reduce the risk of injury to normal coronary arteries, ensure secure closure of both inflow and outflow vessels associated with the aneurysm, and consequently minimize intraoperative blood loss.

## References

[1] Swaye PS, Fisher LD, Litwin P (1983). Aneurysmal coronary artery disease. Circulation.

[2] Li D, Wu Q, Sun L (2005). Surgical treatment of giant coronary artery aneurysm. J Thorac Cardiovasc Surg.

[3] Lima B, Varma SK, Lowe JE (2006). Nonsurgical management of left main coronary artery aneurysms: report of 2 cases and review of the literature. Tex Heart Inst J.

[4] Syed M, Lesch M (1997). Coronary artery aneurysm: a review. Prog Cardiovasc Dis.

[5] Wan S, LeClerc JL, Vachiery JL (1996). Cardiac tamponade due to spontaneous rupture of right coronary artery aneurysm. Ann Thorac Surg.

[6] Kawsara A, Núñez Gil IJ, Alqahtani F, Moreland J, Rihal CS, Alkhouli M (2018). Management of coronary artery aneurysms. JACC Cardiovasc Interv.

[7] Baman TS, Cole JH, Devireddy CM, Sperling LS (2004). Risk factors and outcomes in patients with coronary artery aneurysms. Am J Cardiol.

[8] Konno S, Endo M (1973). Congenital coronary disease (Article in Japanese). Kokyu To Junkan.

[9] Sekine Y, Kitano M, Akimoto T, Matsuda K (2007). The saccular coronary aneurysm associated with coronary artery to pulmonary artery fistulae (Article in Japanese). Kyobu Geka.

[10] McCrindle BW, Rowley AH, Newburger JW (2017). Diagnosis, treatment, and long-term management of Kawasaki disease: a scientific statement for health professionals from the American Heart Association. Circulation.

